# Structural racism in precision medicine: leaving no one behind

**DOI:** 10.1186/s12910-020-0457-8

**Published:** 2020-02-19

**Authors:** Lester Darryl Geneviève, Andrea Martani, David Shaw, Bernice Simone Elger, Tenzin Wangmo

**Affiliations:** 1 Institute for Biomedical Ethics, University of Basel, Basel, Switzerland; 2 Care and Public Health Research Institute, Maastricht University, Maastricht, the Netherlands; 3 University Center of Legal Medicine, University of Geneva, Geneva, Switzerland

**Keywords:** Precision Medicine, Racial bias, Racial discrimination, Healthcare inequalities, Social justice, Ethics, research

## Abstract

**Background:**

Precision medicine (PM) is an emerging approach to individualized care. It aims to help physicians better comprehend and predict the needs of their patients while effectively adopting in a timely manner the most suitable treatment by promoting the sharing of health data and the implementation of learning healthcare systems. Alongside its promises, PM also entails the risk of exacerbating healthcare inequalities, in particular between ethnoracial groups. One often-neglected underlying reason why this might happen is the impact of structural racism on PM initiatives. Raising awareness as to how structural racism can influence PM initiatives is paramount to avoid that PM ends up reproducing the pre-existing health inequalities between different ethnoracial groups and contributing to the loss of trust in healthcare by minority groups.

**Main body:**

We analyse three nodes of a process flow where structural racism can affect PM’s implementation. These are: (i) the collection of biased health data during the initial encounter of minority groups with the healthcare system and researchers, (ii) the integration of biased health data for minority groups in PM initiatives and (iii) the influence of structural racism on the deliverables of PM initiatives for minority groups. We underscore that underappreciation of structural racism by stakeholders involved in the PM ecosystem can be at odds with the ambition of ensuring social and racial justice. Potential specific actions related to the analysed nodes are then formulated to help ensure that PM truly adheres to the goal of *leaving no one behind*, as endorsed by member states of the United Nations for the 2030 Agenda for Sustainable Development.

**Conclusion:**

Structural racism has been entrenched in our societies for centuries and it would be naïve to believe that its impacts will not spill over in the era of PM. PM initiatives need to pay special attention to the discriminatory and harmful impacts that structural racism could have on minority groups involved in their respective projects. It is only by acknowledging and discussing the existence of implicit racial biases and trust issues in healthcare and research domains that proper interventions to remedy them can be implemented.

## Background

The *Precision Medicine Initiative* (PMI) working group defines PM as “an approach to disease treatment and prevention that seeks to maximize effectiveness by taking into account individual variability in genes, environment, and lifestyle” [1]. Indeed, technological advances and increasing computing power allow to do a more in-depth characterization of individuals’ variability and hence their predisposition to diseases by considering not only their genomic profiles but other factors, including other omics (e.g. metabolomics), and their environmental and mobile data. The goal of PM is to advance medical and scientific discoveries while offering more tailored, precise and accurate health interventions, which will maximize the health benefits for patients [2, 3]. With such an approach, individual well-being is monitored pro-actively, that is, PM is predictive, personalized, preventive and participatory (in this context, the terms PM, personalized medicine and “P4 medicine” are used interchangeably) [4, 5].

The defining features and goals of PM seem to make it complementary to the scope of health equity as defined by the World Health Organization [6] that “ideally everyone should have a fair opportunity to attain their full health potential and that no one should be disadvantaged from achieving this potential”. In fact, PM is aimed at providing patients with preventive and therapeutic interventions based on their individual needs (e.g. their susceptibility profile to some diseases). On a practical level, the objective of PM initiatives is to help physicians better comprehend and predict the needs of their patients, so that they may adopt the most suitable treatment in a timely manner. This goal is promoted by sharing health data and implementing learning healthcare systems [7]. Given the overall continuity of the clinical objectives, PM should be considered an “evolutionary” rather than a “revolutionary” approach to clinical trials, medicine and clinical care [8]. Indeed, technological advances over the years (e.g. relatively cheap genomic sequencing or tumour profiling) are accelerating scientific discoveries and subsequent market approval of new therapeutics in comparison to conventional means (e.g. by reducing the required number of participants for clinical trials or even the need for a control group) [8].

Alongside its promises, PM also entails the risk of exacerbating health inequalities, in particular between racial and ethnic groups. The fact that PM has a participatory component requires that different racial and ethnic groups trust and actively engage in PM initiatives [9], which is, however, extremely challenging. Minority communities often face discrimination in healthcare and receive poor medical treatment [10]. Outreach to these communities – especially in the research field – has also been characterized by a long history of exploitation, abuse and marginalization [11]. Events like the Tuskegee syphilis experiment [12] or cases like that of Henrietta Lacks [13] are often cited as causes of distrust by minority groups towards healthcare services and involvement in research projects. However, the relatively lower participation rate of minority groups in health research is not simply a matter of distrust and unwillingness [14]. In a review of enrolment decisions of more than 70,000 individuals for participation in health research, Wendler and colleagues [14] have shown that willingness to participate did not differ significantly between ethno-racial groups and argued that underrepresentation of minority populations is more likely due to the research design of the single study or to limited accessibility. Aside from its cause, lower participation of minority groups has also contributed to most genetic databases used for research purposes containing data on participants of predominantly European ancestry [11, 15]. From an analysis of Genome-Wide Association Studies (GWAS) representing 1.7 million samples conducted in 2009, it resulted that 96% of participants were of European ancestry. Seven years later, the same GWAS analysis revealed that racial and ethnic representativeness of the samples still had a long way to go. In spite of the colossal 35 million samples collected, 81% of participants were still of European ancestry [16]. That racial and ethnic minorities are marginalized in the research field has also been underscored by the authors who concluded that “the message being broadcast by the scientific and medical genomics community to the rest of the world is currently a harmful and misleading one: the genomes of European descendants matter the most” [16].

The situation in the healthcare sector is similarly discouraging: a prominent scholar has recently underscored that, with respect to health, “America is Failing its Black Mothers” [17]. For instance, the pregnancy-related mortality ratio in the US during 2011–2016 for black women was 42.4 deaths per 100,000 live births, more than three times higher than for white women [18]. In a report by Amnesty International [19], titled “Deadly Delivery, the maternal health care crisis in the USA”, it was reported that some healthcare providers do not take into account the timely healthcare needs of women of colour and treat them suboptimally or sometimes even try to dissuade them from seeking medical care, which left these women feeling “ignored or treated with disdain by staff”. Therefore, health disparities and pregnancy outcomes of women of colour are influenced by systemic factors that either regulate access to healthcare or influence the quality of care offered to minority groups.

Although much of the research at the intersection of healthcare and race is conducted in the United States [20], the situation of minority groups is likely to be similar in Europe [10]. For instance, a large European study on end-stage renal disease demonstrated that “black and Asian patients were about half as likely to receive a kidney transplant as white patients, a finding that was not explained by differences in cause of kidney failure” [21]. The authors highlighted that disparities, both in terms of mortality on renal replacement therapy and decreased access to renal transplantation, could not be explained completely by the cause of kidney failure, and that other factors such as the socioeconomic, cultural, environmental and even other biological factors were likely to be involved [21]. Moreover, it is worrying that little is known on the epidemiological profile of minority groups in European high-income countries due to their often unfortunate exclusion in epidemiological studies [22]. Their inclusion would contribute to a better understanding of the healthcare inequalities faced by members of these communities. Additionally, this lack of epidemiological data is a missed opportunity in the era of PM. For instance, coupling epidemiological data with genetic data could provide some additional insights on how socio-cultural, economic and environmental factors influence biological pathways in minority groups and contribute to the pathogenesis of certain diseases (e.g. heart disease) [23].

As rightly pointed out by Bayer and Galea [24], PM initiatives tend to focus mostly on individual health rather than considering how social determinants and structural realities (e.g. residential segregation for minority groups) have shaped and are continuing to shape population health. This, coupled with the pre-existing structural problems illustrated above, supports discriminatory actions against minority groups, which altogether increases their vulnerability to adverse health outcomes [19], provoking distrust in the healthcare system, which will precipitate to PM initiatives [25]. Therefore, it is crucial to identify and better understand the underlying systemic factors that jeopardize the trust of minority groups in healthcare professionals, and institutions now dedicated in advancing the goals of PM. Without a trusting relationship between minority groups and PM initiatives, these are unlikely to succeed in their research objectives, as representative collection and integration of health data (from EHRs, tissue samples, etc.) will be compromised [25].

One societal phenomenon that can in part explain such disparities in the quality of healthcare provided to different ethnic and racial groups is racism [26]. According to *Oxford English Dictionary*, the word “racism” is defined as “prejudice, discrimination, or antagonism directed against someone of a different race based on the belief that one’s own race is superior” or “the belief that all members of each race possess characteristics, abilities, or qualities specific to that race, especially so as to distinguish it as inferior or superior to another race or races” [27]. Given the influence that racism still has in healthcare, the marginalization of racial and ethnic minorities might not be the only reason why PM does not stand up to its promise of providing equal chances for all. From our perspective, an underestimated factor whereby PM can contribute to inequality in healthcare and research between different ethnoracial groups is its susceptibility to racism in general and to *structural* racism in particular. The term *structural racism* refers to “ideologies, practices, processes, and institutions that operate at the macro level to produce and reproduce differential access to power and to life opportunities along racial and ethnic lines” [28]. Over centuries, it has been entrenched in numerous countries, influencing the way medicine is taught and practiced as well as the functioning of healthcare institutions [29]. This might help to understand why – although genetic predisposition or unhealthy lifestyle, biological inferiority, socio-economic factors, and medical distrust are put forward as some of the reasons contributing to the persistence of healthcare inequalities between ethnic and racial groups [30, 31] – even when some of such factors are taken into account, these inequalities remain [32]. Furthermore, unhealthy lifestyle and socio-economic factors themselves are in turn partly a product of structural racism and discrimination [30].

Claiming that PM initiatives might be subject to the influence of structural racism might sound controversial, since – theoretically – PM endorses social and racial justice between racial and ethnic groups. For instance, the majority of PM initiatives are implemented with the aim of ensuring ethnic diversity and appropriate ethnoracial representation in their cohorts (e.g. All of Us Research Program, New York University’s Human Project and Project Baseline) [33]. Such measures aspire at ascertaining that no racial or ethnic group is left behind and that every individual, irrespective of his or her racial and ethnic backgrounds, benefits from advances in healthcare. However, in spite of its ambition to promote social and racial justice, PM might nonetheless end up accentuating healthcare inequalities between different racial and ethnic groups if it covertly adopts the existing cultural processes such as identification (racialization, e.g. associating racial stereotypes with some therapeutic options, and stigmatization) and rationalization of health services provided to certain racial groups and ethnic minorities [34, 35]. Therefore, PM might reiterate the current *status quo* in healthcare, where very few racial groups are privileged to the detriment of others, especially if structural racism is not taken into account.

In this debate paper, we discuss the ways in which the implementation of PM might be particularly vulnerable to structural racism in healthcare and research, and forecast its potential impacts in the upcoming era of PM. Specifically, we analyse three nodes in the process flow of PM where structural racism can have an impact. These nodes are part of a process flow: (1) collection of biased health data during the initial encounter of patients with healthcare system and researchers, (2) integration of biased health data for PM initiatives and (3) the influence of structural racism on deliverables of PM initiatives. After analysing the interaction of PM and structural racism, we propose future actions to help make PM initiatives truly adhere to the goal of “Leaving no one behind”, as endorsed by member states of the United Nations for the 2030 Agenda for Sustainable Development [36].

## Main text

### The three nodes of structural racism in precision medicine

In the ecosystem of PM, there seem to be three specific nodes where structural racism can have an impact: the quality of health data collected; the integration of these data in PM initiatives; and the development of new therapeutics, diagnostics or disease prevention strategies. In this context, the concepts of clinical and research data are grouped together under health data, as boundaries between clinical practice and research are blurring in the convergence framework of PM, learning healthcare systems and implementation science [37]. A learning healthcare system would allow the improvement of care over time by collecting data in the clinical encounter and using them to improve the effectiveness and efficiency of current clinical practice, by facilitating the exchange of information between clinical and research sectors [9, 37]. It would thus act as a bridge for the integration of new discoveries made through PM initiatives into routine clinical care, whereas implementation science would be the “catalyst” to such transition by providing strategies to promote the operationalisation of PM initiatives’ new findings [37, 38]. These three nodes are used in the following sections to structure our arguments.

### First node: collection of biased health data during initial encounter with healthcare system and researchers

The first node depicts the initial encounter between minority populations and healthcare providers and/or researchers, which leads to the production of biased health data, collected (among others) in electronic health records (EHR), biobanks or different research data banks [9]. For the first node, there are two distinct aspects, which need to be carefully considered. Firstly, minority groups are under-represented in current health services and research datasets, due to unequal access to healthcare and clinical studies [14]. Such underrepresentation can negatively affect the quality of health services provided to their members, since they might be treated according to guidelines informed by biased data – in the form of data that disproportionately represent people of the majority ethnic or racial group [33]. For example, PM is spearheading the fight against certain types of cancer [2], owing to technological advances made in genomics (e.g. with the advent of next-generation sequencing allowing the identification of a huge number of variants [39]), cancer biology and other relevant fields. It thus provides a more molecular-based and individualized approach to dealing with both primary and recurrent/metastatic tumours [40]. However, there are numerous barriers hindering the participation of minority groups to genetic testing for evaluating cancer risk [41]. Without enough genetic data for some minority populations, it will be almost impossible to distinguish pathological from benign variants in these subgroups, and consequently, evaluating their risks of developing a certain cancer type might be compromised. Therefore, minority groups at high-risk of developing a disease will not benefit from high quality disease preventive measures [31], even if granted access to similar a treatment to that offered to the majority group.

Secondly, minority groups are more susceptible to receive suboptimal care due to implicit provider bias in healthcare, which also feeds into the problem of biased health data. Indeed, it is known that healthcare providers, irrespective of their specialization fields or levels of experience, suffer from an implicit racial or ethnic bias when dealing with people of colour [42]. Such bias negatively affects their ability to provide efficient health services to minority groups, due to uncontrolled thoughts or feelings that influence their clinical judgement [42, 43]. For instance, a 2016 US study showed that medical students and residents held false beliefs concerning biological differences between black and white people, which negatively influenced their assessment of pain and treatment recommendations in people of colour [44]. Similarly, another study showed that black children were prescribed fewer antibiotics than their white counterparts when examined by the same physician [45]. In the same vein, a study found that healthcare providers in US emergency departments have a high implicit preference for non-Hispanic white people over the American Indian community [46]. Hence, it is clear that in healthcare systems where most professionals are of Caucasian origin, people of colour are at risk of not being given equal access and a level of care comparable to that offered to patients of Caucasian origin. Given these premises, it is probable that even if new individualized treatments are available to people of colour, PM initiatives will fall short of their goals.

Indeed, equal access to individualized prevention and treatment might be compromised by unconscious racial bias already existing in the healthcare context. Due to these negative implicit racial stereotypes [47], healthcare providers might not prescribe new therapeutic drugs to these communities or might treat them suboptimally. Moreover, this “aversive racism” (i.e. having a high degree of implicit bias and a relatively low degree of explicit bias) during medical encounters, which is under-recognized and habitually unintended, leads minority groups to respond more negatively to physicians [48]. Aversive racism thereby undermines patient’s trust due to lower perceived quality of care, poor doctor-patient communication as well as a loss of interest in joint decision-making [48, 49]. These are all detrimental to the goals of PM initiatives, since they lead to biased data being produced for minority groups, who, in turn, are less likely to engage in research activities. Although biased data originate predominantly from subjective interpretation (e.g. biased clinical evaluation by physicians) rather than objective measurements (e.g. results of an MRI scan, blood tests), it nonetheless remains possible that objective data on minority groups are not being captured optimally due to biased clinical evaluation of their medical conditions.

Since PM initiatives gather data from both new and existing sources (e.g. electronic health records, biobanks, etc.), Ferryman and Pitcan [33] highlighted that, in the era of PM, “it is important to recognize the potential limitations within these data today that come from historical legacies of bias and discrimination”. On top of that, we argue that it is also important for PM initiatives to better understand the limitations of new data collected by physicians or researchers involved in PM initiatives that belong to the majority group. Indeed, the iterative nature of PM initiatives and learning healthcare systems implies that data are gathered continuously to generate new insights into individuals’ health, which are thereafter implemented in practice for better-individualized prevention and treatment. However, if minority groups suffer from racially discriminatory actions in clinical practice and are offered less effective healthcare interventions due to biased non-representative data, the chain of healthcare improvements based on reliable routinely collected clinical data may be compromised from its very start.

Due to past betrayals of trust, minority groups might be reluctant to engage with their healthcare system, leading not only to a lack of interests in PM activities but also limited data representativeness from these groups [33]. It is crucial to understand the expectations and fears of minority groups regarding their participation in PM initiatives. For instance, a recent study showed that minority groups also fear that, by participating to PM initiatives, results of these initiatives might unwillingly contribute to further racial discrimination from either the healthcare system (e.g. being denied access to treatment because it is specific to an ethnic/racial group) or by their healthcare insurers and employers (e.g. loss of employment opportunities or higher insurance premiums) [25]. This altogether has negative repercussions on the quality of health data collection efforts to provide evidence-based healthcare and on the development of accurate clinical guidelines or treatments for these communities [33].

The production of biased health data for minority groups leads us to the second node of the process flow.

### Second node: integration of biased health data for PM initiatives

The second node characterizes the integration of biased health data from minority populations into PM initiatives, leading to their faulty interpretation and thus to misuse in scientific research and clinical practice [33]. With respect to this node, it is crucial to acknowledge the twofold potential damage resulting from biased PM initiatives for minority groups. Firstly, health data have always been prone to historical biases and minority groups are already paying a high price for them. For instance, current clinical guidelines are largely developed from cohorts of white men, whose risks factors for developing a particular disease could be very different from men (and women) belonging to minority populations. Therefore, this sampling bias implies that the threshold required to justify certain medical interventions or disease prevention strategies would differ based on an individual’s racial or ethnic background [33]. One concrete example that was widely covered in the literature is the *Framingham Coronary Heart Disease Risk Functions*, a risk assessment score used for the primary prevention of coronary heart disease (CHD). The *Framingham Risk Score* was originally developed from a population of principally white cohorts in the USA to predict the risk of CHD and subsequent appropriate preventive measures. It was shown to overestimate the risks of cardiovascular diseases not only in some minority groups (e.g. Hispanic and Japanese American men [50]), but also in some European (e.g. Germany [51]) and non-European countries (e.g. China [52]), thereby highlighting the need for recalibration. Until eligibility for interventions and interventions themselves are calibrated, it is thus probable that data required for the good functioning of PM initiatives are not being captured for minority groups. Secondly, the former problem (historical bias), coupled with structural racism, could be amplified with the increasing use of artificial intelligence (AI) technologies to assist physicians and researchers in their routine work [33].

The application of AI technologies is rapidly increasing in the healthcare sector [53], and according to Ferryman and Pitman [33], AI is also a necessary feature of PM due to the increasing availability of big health data sources. Indeed, AI technologies are considered to be one of the solutions to help researchers and physicians interpret the ever-growing amount of health data produced on a daily basis, which already greatly exceed physicians’ analytical capabilities [53]. However, there is increasing concern that these AI technologies are hugely dependent on the data that they are trained with and can subsequently aggravate societal biases present in the training databases [54]. Whereas decisions by healthcare providers or researchers might be only *intermittently* influenced by racial bias, decisions made by machine learning algorithms will be *systematically* biased every time the latter are used, leading to more discrimination against minority groups and to a much larger scale [55]. Indeed, how historical bias in the training datasets and hence in algorithmic decisions can lead to more discrimination is perfectly illustrated by the case of the AI tool named COMPAS (Correctional Offender Management Profiling for Alternative Sanctions) used in the US judicial sector. COMPAS was a software designed to support judicial decision-making concerning potential recidivism of offenders. It assigned probability scores to defendants on whether they were likely to break the law within 2 years after being released from prison. COMPAS was shown to be biased against black offenders due to presumed historical bias in the data, an element which led to more black people being kept in prison rather than being released just because of their ethnicity [56]. This case was particularly interesting as one could even argue that the bias of the software against black people was not immediately perceived by the judges using the AI tool because racial and ethnic prejudices are so deeply rooted and implicit that they easily go unnoticed. In the same vein, it would not be surprising if historical bias in health datasets used in the training of AI technologies for PM initiatives, coupled with structural racism, ended up reproducing existing healthcare inequalities between racial and ethnic groups. If so, physicians – just like judges in the case of COMPAS – would be very unlikely to identify flawed medical decision-making induced by AI, because of their pre-existing prejudices.

On top of biased medical decision-making, AI may also have a negative impact on the recruitment of people of colour in clinical trials. Clinical trials have traditionally been known to be time- and resource-consuming, with difficulties in “matching the right trial with the right patient”, but AI has been forecasted to provide the solution to this by automating the whole clinical trial matching through available health data sources [57]. For instance, DEEP 6 AI is a software company based in the US that analyses both structured and unstructured data using machine learning, natural language processing and medical ontologies with the aim of matching eligible patients to potential clinical trials in a timely manner [58]. Another example comes from Microsoft, who, as part of their Microsoft Healthcare Bot initiative [59], use machine reading to assign suitable patients to clinical trials with the aim of streamlining the whole recruitment process [60]. Similar to biased medical decision-making, we argue that the use of AI technologies in automatically assigning patients to clinical trials in the PM era may also be negatively influenced by historical bias in the health datasets (e.g. EHRs) [33] and by structural racism. If not properly designed, these AI technologies could exacerbate health inequalities between minority and majority groups by either excluding or limiting the eligibility of people of colour to participate in certain studies. For instance, Obermeyer and Mullainathan [61] discovered how an algorithm used in US healthcare on over 70 million patients was racially biased against black people. The algorithm reduced the chances of black people being enrolled in the “care management program”, and the culprit was not the training datasets *per se* but rather the inappropriate choice of labels (e.g. healthcare costs) which did not provide the full picture regarding the health of black people [61]. Another example came from the University of Chicago hospital system, where researchers found that if postal codes had been used in their machine-learning algorithm to optimize hospital resources, resources available for black people would have been diverted towards “wealthy white people, exacerbating existing biases in the system” [62]. As residential segregation is also a known consequence of structural racism [63], this shows how structural racism can have many repercussions on algorithmic decisions in the healthcare system.

### Third node: influence of structural racism on deliverables of PM initiatives

The third node refers to the uptake of new disease prevention strategies, diagnostics and therapeutics from PM initiatives into the cycle of learning healthcare frameworks. The goal of a learning healthcare system is to provide better care to individuals over time by continuously collecting clinical encounter data and using them to develop strategies to improve the quality of care offered to patients. It thus provides a unique opportunity for findings of PM initiatives to be implemented in the routine clinical life cycle [37]. With respect to this node, the risk of racial discrimination is due to the potentially discriminatory effects of feeding biased data into a learning healthcare framework, especially because current healthcare systems are already designed and built for patients of the majority group [10] and are consequently not customized for minorities in terms of their reduced access to care. There are numerous reasons for reduced access to care, including (1) the fact that minority groups are sometimes unable to pay for health services due to lower health insurance coverage, (2) medical distrust as a result of previous racially discriminatory actions or perceived racism (which in itself is an additional detrimental stressor to the health of minority groups [26]) that delay or prevent access for treatment, and (3) the geographical variation in healthcare quality offered to minority groups [64–66]. In a learning healthcare framework, reduced access to healthcare implies the loss of important clinical encounter data from minority groups due to reduced or delayed contacts with physicians [65], which would normally help to improve the monitoring of disease evolution and subsequent appropriate treatment options. This bias induced through “invisibility” - caused by insufficient data or incomplete datasets on minority groups - can potentially lead to adverse and discriminatory health outcomes as easily as overtly flawed data [33].

Over the past few years, there has been a real commitment from pharmaceutical companies to advance the goals set by PM initiatives by producing new personalized medicines. In 2018, the Center for Drug Evaluation and Research of the FDA approved a record number of 25 new personalized therapeutics, which represented 42% of the total number of drug approvals for that year [67]. In this respect, another important aspect, in terms of minority groups’ access to adequate healthcare, may be the lack of interest in developing new therapeutic options for diseases more prevalent in minority groups, due to structural racism embedded in the world of research and in drug development. From our perspective, Farooq and Strouse [68] gave an excellent example of potential racial bias in research and drug development by comparing two distinct diseases, each one predominantly affecting a different racial group.

*Cystic fibrosis*, affecting predominantly white populations, is an autosomal recessive disease resulting from a defect in the gene encoding for the chloride channel, CFTR (cystic fibrosis transmembrane conductance regulator). This alteration leads to pulmonary complications such as chronic bacterial infections, bronchiectasis and pulmonary fibrosis [69]. S*ickle-cell disease* (SCD), on the contrary, mainly affects people of colour. Globally, SCD is one of the most severe blood conditions, caused by a mutation in the beta globin gene, which leads to the production of sickle globin instead of beta globin, a component necessary for the production of normal haemoglobin. This genetic mutation causes the occlusion of blood vessels and haemolytic anaemia, resulting in many complications such as premature death, acute chest syndrome or cerebrovascular disease (e.g. stroke) [70]. Although both conditions have similar disease severity, and a lower percentage of patients in the United States suffers from *cystic fibrosis* in comparison to SCD, Farooq and Strouse [68] demonstrated that there are wide disparities in how funding was allocated for research by the National Institutes of Health (NIH) and private Foundations to study the two diseases. Moreover, research productivity in terms of PubMed-indexed articles and drug approvals were significantly higher for *cystic fibrosis* than for SCD, in spite that both diseases have similar numbers of clinical trials [68].

With reference to the last five progress reports (2014–2018) of the *Personalized Medicine Coalition* [67, 71–74], we observed the same trend regarding FDA approval of personalized drugs for the two diseases. Two personalized drugs for *cystic fibrosis* were approved in this time span: Orkambi (*ivacaftor* and *lumacaftor*; 2015) and Symdeko (*ivacaftor* and *tezacaftor*; 2018); but none for SCD. Therefore, it remains imperative to underline that although minority groups could actively engage in PM initiatives by voluntarily contributing their data for research, the research and pharmaceutical sector might be biased in improving or finding new diagnostics and therapeutics for diseases prevalent in the white population. Such discrimination is partly caused by financial interests of pharmaceutical companies, which prioritize drug development for western market, as the countries can afford the high prices of developed drugs. In 2014, the ex CEO of Bayer, Marijn Dekkers, raised a lot of controversy when he declared that his company only produced cancer medication for “western patients who can afford it” and not for the Indian market, a statement condemned massively by *Médecins Sans Frontières* [75]. Therefore, if PM wants to achieve its equity goals and thereby safeguards the trust and long-term engagement of minority groups, it is paramount to ensure that members of minority groups see the clear benefits that their communities will get in return for their participation to PM activities [25].

### Connecting the nodes – some future actions

**Fig. 1:**
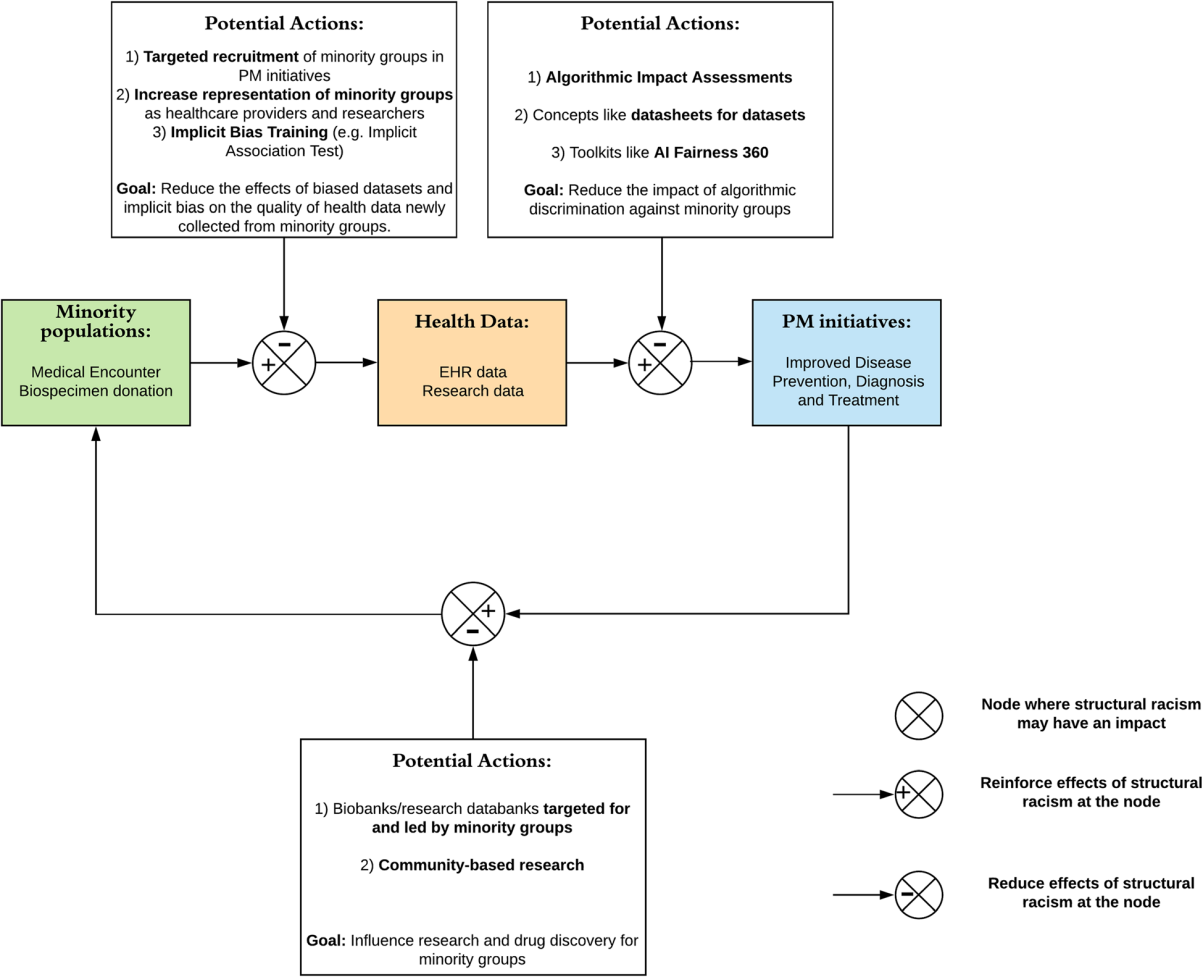
Potential actions to reduce the cascading effects of structural racism on the quality of health data collection, integration and deliverables in precision medicine initiatives

It is to be expected that the mentioned deleterious effects of structural racism will be reinforced over time in PM initiatives, due to the iterative data–exchange process between research and the clinical sector. Therefore, it is paramount to consider the impacts of structural racism at the very outset of PM initiatives, in order to prevent continued discriminatory treatment of minority groups in research and during clinical care. It is also important to recognize that trust [76] and engagement of minority groups in PM initiatives need to be safeguarded for PM to achieve its full potential. In the previous sections, we explored the nodes where structural racism could affect the implementation of PM initiatives, and forecasted that bias induced by structural racism in health datasets can have cascading deleterious effects on the health of minority groups. In the following sections, we recommend some potential actions that can help ensure those negative effects are mitigated (Fig. 1).

### Potential actions to reduce the impact of structural racism at node 1

Regarding the limited representation of minority groups in current health and research databases, some PM initiatives are already taking actions to address these issues. For instance, the *All of Us Research Program* [77] has been prioritizing minority groups for the collection of biospecimens and physical examinations. In a special report published in *NEJM,* the investigators stated that, as from July 2019, “more than 80% of these participants [over 175,000 participants donated their biospecimens] are from groups that have been historically underrepresented in biomedical research” [77]. This represents a huge step forward towards improving data representativeness for minority groups and ensuring that they will be offered healthcare interventions that are adequately tailored to their ‘real’ needs and not extrapolated from non-representative data. In contrast, the *UK biobank*, a valuable resource for PM [78], with over 500,000 study participants recruited over 2006 to 2010 [79], has adopted a different approach. Although explicitly acknowledging the limited generalizability of their data to the general population of the United Kingdom [80], the *UK biobank* relies on the large cohort size to cater for selection bias and the resulting limited data representativeness for *accurately* assessing “exposure-disease relationships” [81]. In this regard, we could not agree more with the correspondence from Keyes and Westreich [82], where it was argued that inferences derived from large sample sizes can also be skewed (to the detriment of external validity) and such aspects should be “taken more seriously in the UK biobank and other large data resources”. Therefore, we deem more appropriate to have better targeted recruitment and interventions, like those carried out by the *All of Us Research Program*, which would help reduce selection bias and limited data representativeness of minority groups in PM initiatives.

Aside from tackling the problem of representativeness at the institutional level, actions needs to be taken also at the individual and professional level. According to 2017 statistics, the great majority of physicians and surgeons in the United States were white, accounting for 69.8% of the workforce, followed by Asian (21.1%), black (5.8%) and other minority groups [83]. As put forward by Cohan in a recent *NEJM* article [84], “… health care is not safe for people of colour as long as the overwhelming majority of U.S. physicians are white and we avoid examining where racism lives within us and how it lives through us”. Therefore, we can reasonably argue that one of the reasons underlying the normalization of discriminatory actions against people of colour in healthcare is the lack of racial and ethnic representativeness in healthcare professions and the under-recognition of the impacts of structural racism by white physicians. The EU parliament has recently (26/03/2019) passed a resolution where it invites other “European institutions to adopt a workforce diversity and inclusion strategy that establishes a strategic plan for the participation of ethnic and racial minorities in their workforce” [85]. This commitment may help to address this problem in Europe. However, it is important not only to increase the percentage of minority groups as physicians, researchers and in other healthcare professions, but also to request white physicians to see their routine clinical work in a new light when dealing with people of colour. A good option to assess their degree of implicit racial bias against minority groups is through the *Implicit Association Test* developed by Project Implicit [86], which aims to educate the population about unconscious biases. Data from Project Implicit have already been used to reveal how racial prejudice negatively influences birth outcomes for black women in the United States [87], or even the pervasiveness of implicit prejudices against the lesbian, gay, bisexual, and transgender (LGBT) community among healthcare providers [88]. Such tests could help white physicians to better identify and subsequently question the biased choice of treatment for minority groups. 

These different measures will help to ensure that hospitals, research institutions and other similar structures function on principles, values and foundations which are representative of the ethnic and racial make-up of their society [89]. These actions will also contribute to improving the quality of health data collected on minority groups, since they will hopefully reduce racial discriminatory actions and restore trust. Gaining the trust of minority groups and ensuring that data collection is less affected by bias introduced by structural racism will forward the promised health benefits of PM initiatives to help bridge the health gaps between racial and ethnic groups.

### Potential actions to reduce the impact of structural racism at node 2

The issues surrounding biased algorithmic decisions has not left lawmakers indifferent [90], in particular following recent notable big tech scandals (e.g. the Cambridge Analytica Affair [91] or the fact that the Department of Housing and Urban Development in the US is suing Facebook over discrimination in housing-related advertising [92]). To tackle some of these technology-related issues, a new bill has been recently introduced in both Houses of the US Congress, the *Algorithmic Accountability Act 2019,* which aims at ensuring fairer and non-discriminatory algorithmic decisions. Although representing an important step in the fight against algorithmic discrimination, it has been underscored that this bill seems to be lacking in three important aspects: (1) at the level of enforcement, it relies on the Federal Trade Commission, which, as an agency, rarely enforces its settlements with privacy violators; (2) at the level of impact assessments, it lacks an avenue for diverse public participation, in particular from affected communities; and (3), also at the level of impact assessments, it does not provide for them to be made public [90]. One solution to these issues could come from the implementation of *Algorithmic Impact Assessments* (AIA) for public agencies, to ensure that *automated decision systems* are not only assessed by involved stakeholders, but also by members of communities affected by these systems [93]. Within the AIA framework, the concerned agency would need to publicly disclose its definition of *automated decision system*, any assessments and external reviews made on the potential impacts of the system before its procurement, and the public would then be allowed to comment on the system and clarify its concerns with the agency. Additionally, the government would have the duty to ensure that the rights of affected individuals are respected by providing improved due process tools, in cases where an agency has not corrected a biased system. Such measures would hold the concerned agency accountable while safeguarding against unlawful discrimination or the non-respect of rights of affected communities [93].

Another solution put forward by Gebru and colleagues [54] is the concept of *datasheets for datasets*, which would help tackle the issues surrounding biases in training datasets for machine learning communities. According to the authors, each dataset should be accompanied by a datasheet explaining the characteristics of the dataset (e.g. motivation, composition, collection process, etc.). These datasheets could potentially address the biases in training datasets for machine learning processes by increasing not only transparency but also accountability within machine learning communities [54]. Researchers, tech companies, and physicians would thus be able to make a more informed choice in the selection of adequate datasets for a given task and therefore reduce the impact of biases against minority groups.

Assessment and corrective measures can be taken against algorithmic discrimination with either the training dataset, the learning procedure (i.e. the classifier) or the predictions of the AI tool. In this regard, IBM has proposed the *AI Fairness 360* (AIF360), an open source toolkit aimed to “promote a deeper understanding of fairness metrics and mitigation techniques; to enable an open common platform for fairness researchers and industry practitioners to share and benchmark their algorithms; and to help facilitate the transition of fairness research algorithms to use in an industrial setting” [94]. Depending on where the intervention is needed to avoid algorithmic bias in the AI cycle, AIF360 proposes three approaches, namely *pre-processing* (actions needed on the training dataset), *in-processing* (actions needed on the classifier) and *post-processing* (actions needed to correct predictions) bias mitigation algorithms [94]. Regardless of the instrument used, education on strategies to check and mitigate algorithmic bias in their tools could be extremely beneficial for AI developers active in the field of PM.

### Potential actions to reduce the impact of structural racism at node 3

Another factor that might undermine the good health intentions of PM initiatives towards minority groups is the limited access to healthcare and new therapeutics. A first fundamental step to try remedy this situation is to intervene in the processes of creation and development of biobanks. According to Shaw and colleagues [95], “a biobank is any collection of human biological samples and linked data that is to be used for research”. These, together with databanks, are globally regarded as essential research infrastructures for PM, allowing the collection of health data from large cohorts, and deriving “wisdom from crowds” to deliver individualized treatment [78]. However, in biobanks and databanks, there is often an underrepresentation of minority groups. This is not only the result of recruitment difficulties but also of the deliberate exclusion of these groups by scientists, as their inclusion in studies will lead to confounding results due to genetic variation [96]. The unfortunate consequence of such exclusion or underrepresentation is the exacerbation of healthcare inequalities between racial and ethnic groups, because it is more unlikely that treatments tailored to their needs are discovered.

To tackle this issue, efforts made to introduce biobanks specific to and led by minority groups should be praised and strongly encouraged. One such example is the BRAICELET project (Bio-Repository for American Indian Capacity, Education, Law, Economics and Technology), which aims to reduce health inequalities “through the establishment of a first-of-its-kind American Indian Biobank” [97]. In the BRAICELET project, American Indian communities are allowed to “lead collaborations with universities and research institutes across the nation to find culturally and real-time solutions to issues of disparity affecting American Indian communities” [97], enabling the implementation of programs that are tailored to the needs of these indigenous communities. Similarly, the National Institutes of Health and the Wellcome Trust jointly funded a large-scale initiative, called *Human Heredity and Health in Africa* (H3Africa) to allow the implementation of PM in the continent. H3Africa seeks to facilitate research on diseases affecting African populations by gathering genetic and environmental data on tens of thousands of participants [98]. The data gathered by H3Africa will be used to influence research in the field of pharmacogenomics, where African communities have long been marginalized, with the goal of discovering drugs most susceptible to benefit the health of African populations [98]. Some minority groups also view community-based research not only as being more valuable to their communities, but also as a means of motivating them to participate in activities of PM initiatives [25].

## Conclusions

Structural racism has been entrenched in our societies for centuries and it would be naïve to believe that its impacts will not spill over in the era of PM. In this perspective, PM initiatives around the world should pay particular attention to the potential impacts that structural racism could have on their respective projects, and consider the three nodes analysed in this paper. PM initiatives should embrace the responsibility to mitigate the described impacts of structural racism, in particular those impacts upon which they have direct control. Therefore, careful consideration needs to be given to the choice of health datasets used in their projects to limit racial biases (e.g. the *datasheet for datasets* concept can be a good starting point) and their collaborators (e.g. physicians, researchers and technology developers) need to be better informed about the detrimental and insidious impacts of structural racism on their activities. For instance, the *Implicit Association Test* could allow physicians to reflect upon their routine clinical practice to identify situations where their attitudes and medical decisions for minority groups might have been influenced by unconscious biases and promptly try to remedy the situation by sensitizing themselves to the cultural values and perspectives of minority groups. These initiatives should also encourage the implementation of specific biobanks and other research databanks targeted for minority groups, with the mandatory inclusion of members of these communities at the management level, to ensure that scientific discoveries are stirred towards improving or finding new treatment for diseases affecting predominantly minority groups (e.g. through community-based research). Although not falling directly under their control, PM initiatives should also encourage and lobby for an adequate representation of ethnic minorities in healthcare professions so that the quality of health data collected for minority groups is improved, with the aim of reducing healthcare inequalities between racial and ethnic groups.

Above all, we believe that it is only by openly acknowledging and discussing the existence of implicit racial biases and trust issues in the healthcare and research domains that proper interventions can be implemented against structural racism. PM could offer a unique opportunity to bridge some of the long-standing racial gaps in healthcare and research. It, however, requires that the deleterious impacts of structural racism are carefully considered and addressed during the implementation of PM initiatives. This will help to prevent the reproduction and perpetuation of the current healthcare inequalities between different ethno-racial groups.

## Data Availability

Not applicable.

## Abbreviations

AI: Artificial intelligence
AIA: Algorithmic Impact Assessments
BRAICELET: Bio-Repository for American Indian Capacity, Education, Law, Economics and Technology
COMPAS: Correctional Offender Management Profiling for Alternative Sanctions
EHR: Electronic Health Record
GWAS: Genome-Wide Association Studies
H3Africa: Human Heredity and Health in Africa
LGBT: lesbian, gay, bisexual, and transgender
PM: Precision Medicine
SCD: Sickle-cell disease
